# Interferon-beta-induced changes in neuroimaging phenotypes of appetitive motivation and reactivity to emotional salience

**DOI:** 10.1016/j.nicl.2019.102020

**Published:** 2019-11-14

**Authors:** Christoph Coch, Roberto Viviani, Jörg Breitfeld, Katrin Münzer, Juliane Dassler-Plencker, Stefan Holdenrieder, Martin Coenen, Michael Steffens, Marcus Müller, Gunther Hartmann, Julia Stingl

**Affiliations:** a Institute of Clinical Chemistry and Clinical Pharmacology, University of Bonn, Sigmund-Freud-Str. 25, 53127 Bonn, Germany; b Institute of Psychology, University of Innsbruck, Innrain 52, 6020 Innsbruck, Austria; c Psychiatry and Psychotherapy Clinic III, University of Ulm, Leimgrubenweg 12, 89075 Ulm, Germany; d Department of Research, Federal Institute for Drugs and Medical Devices Kurt-Georg-Kiesinger-Allee 3, 53175 Bonn; e Department of Neurology, University of Bonn, Sigmund-Freud-Str. 25, 53127 Bonn, Germany; f Institute of Clinical Pharmacology, University Hospital of RWTH Aachen, Wendlingweg 2, 52074 Aachen, Germany

## Abstract

Treatment with interferon (IFN) has been associated with depressive side effects. Previous neuroimaging studies have provided information about changes in brain activation patterns in patients under treatment with IFN-alpha, but the effect of other IFNs, or the role of the underlying disease, has yet to be clarified. In the present fMRI study, we looked at brain changes after 8 days of IFN-beta treatment in *N* = =17 healthy volunteers, thus avoiding the possible confound of the effects of underlying pathology in studies of IFN-treated patients with neurological or other medical disorders. We followed a symptom dimensional approach by simultaneously investigating two distinct symptom domains of depressiveness: negative affect (amygdala) and appetitive motivation (ventral striatum). In these early phases of IFN treatment we detected a selective change in neural substrates of appetitive motivation, consistent with the predominant symptomatic change recorded in psychopathology ratings. In contrast, the fMRI phenotype of negative affect, which is known to characterize disorders of affect involving anxiety and depressiveness as well as individual vulnerability to depression, was unchanged after treatment. These findings suggest that IFN may induce an affective syndrome through a mechanism involving down-regulation of appetitive motivation.

## Highlights

• Our study highlights the use of neuroimaging methods to detect and analyze therapy associated side effects, and the main finding of our study is that interferon, known to induce depression as adverse drug effect, leads to selective blunting of appetitive rather than affective stimuli.
• Our study shows the utility of sophisticated imaging methods to assess and clarify the nature of subtle drug effects on emotional and cognitive behavior.
• We think that the use of neuroimaging methods for the prediction and detection of off-target drug effects on mental health is an important topic in personalized medicine.

## 1 Introduction

It is well accepted that infections lead to behavioural changes so that the affected individual favours resting to support the healing process. This behaviour is called ‘sickness behaviour’ and is characterized by social avoidance, motivational blunting, inactivity and fatigue (Dantzer et al., 2008). These symptoms show considerable overlap with those of depressive disorder. This relationship is buttressed by proposals that inflammatory or immune activation states constitute a contributing pathogenetic mechanism of depression. This process, also called “cytokine induced mood-disorder” can be induced by the systemic release of inflammatory cytokines including tumor necrosis factor alpha (TNFα), interleukin-6 (IL-6) and interleukin-1 beta (IL-1β) (Raison et al., 2009).

In addition to inflammatory cytokines, it has been claimed that the main antiviral cytokines, the type I IFNs (consisting of IFNalpha and IFN-beta) induced in the course of viral infections by activation of innate immune receptors or given as drug treatment may also lead to sickness behaviour and depression with significant impact on the well-being of the patient and the feasibility of the treatment (Smith et al., 2015).

IFN-alpha is associated with the development of symptoms of major depressive disorder in approximately one third of patients with multiple sclerosis (Vattakatuchery et al., 2011). In hepatitis C patients under IFN-alpha treatment, inflammatory activation is accompanied by fatigue, motivational impairments, and mood changes (Dowell et al., 2016). These psychopathological side effects start with early changes in fatigue and motivation that occur after hours of the first type I IFN injections, and can mound into the affective symptoms of a full depression after 4 weeks of treatment (Capuron et al., 2002; McNutt et al., 2012). As IFN-beta is known to activate the common type I IFN receptor even in lower doses than IFN-alpha, a similar effect by IFN-beta can be assumed. For IFN-beta, which is routinely used in multiple sclerosis patients, sickness behaviour and depressive symptoms have also been reported (Alba Pale et al., 2017).

The mechanisms by which type I IFNs induce sickness behaviour and depression-like side effects likely involve inflammatory or cytokine-mediated immuno-activation. Cytokines are thought to affect neurotransmitter-mediated behavioural and cognitive phenotypes related to depressive psychopathology (Capuron et al., 2002; Miller et al., 2013; Schafer and Schwaiger, 2003). Recently, a mechanism of how type I IFN triggers sickness behaviour was described in the mouse model suggesting that type I IFN induces CXCL10 (C-X-C motif chemokine 10) in endothelial cells of the blood brain barrier, which in turn impairs the synaptic plasticity leading to the behavioural changes (Blank et al., 2016).

Applying a symptom-dimensional approach, we wanted to investigate phenotypes of depressive psychopathology occurring in healthy volunteers in the early phases of IFN-beta treatment in a functional neuroimaging study (fMRI). The participants were undergoing a study on cytokine responses upon administration of an IFN stimulus for eight consecutive days (Coenen et al., 2015). Two fMRI phenotypes were chosen as representative of two known important aspects of depressive functioning: lack of sensitivity to reward (impairment in appetitive motivation, leading to an anhedonic symptom domain, (Treadway and Zald, 2011)) and hyperreactivity to negative emotional stimuli (Siegle et al., 2002; Stuhrmann et al., 2011; Whalen et al., 2002). These two fMRI phenotypes refer to assessment of ventral striatal activation during anticipation of reward (Abler et al., 2006), and of amygdala activity when exposed to highly arousing negative emotional stimuli (Whalen et al., 2002).

By simultaneously assessing two distinct phenotypes in the early phases of IFN-beta treatment, our intent was to increase the specificity of the characterization of the development of its depressive side effects.

## 2 Methods

### 2.1 Study design

Functional MRI measurements were undertaken as part of the “Immune- and miRNA-response to recombinant IFN-beta in healthy volunteers and patients with relapsing remitting multiple sclerosis” clinical study (trial short title: RESI), listed under EudraCT-number “2012-005475-13” in the EU Clinical Trials Register database (Coenen et al., 2015). The clinical study was conducted at the phase I Unit and the Clinical Study Core Unit of the University Hospital Bonn in accordance with the ICH Guideline for Good Clinical Practice, the relevant national regulations and the Declaration of Helsinki. The study was approved by the ethical review board of the University Bonn.

Of the 18 volunteers recruited for the study, one participant discontinued intervention due to intolerable side effects (high fever, heavy pain and chills). The remaining study cohort consisted of *n* = =7 men and *n* = =10 women with an average age of 26.5 ± 4.9 years (age range, 18–65, see Table 1). Inclusion criteria were physical (as documented by a physical examination and laboratory testing) and mental health (assessed through the Mini-international neuropsychiatric interview, MINI, (Sheehan et al., 1998)). Laboratory parameters covered adequate function of liver (bilirubin, alanine transaminase, aspartate transaminase), kidney (creatinine), thyroid gland (thyroid-stimulating hormone), bone marrow (white blood cells, granulocytes, platelets, hemoglobin) and blood clotting (prothrombin time, partial thromboplastin time). Additionally, female participants obtained a negative serum pregnancy test prior to treatment start and were instructed to use an approved contraceptive method (Pearl index < 1%) during and for 3 months after the trial. Exclusion criteria were abuse of alcohol, drugs or medication as well as a present co-medication with corticosteroids, and a pronounced fear of blood drawings. Furthermore, participants with a known potential allergy/hypersensitivity to IFNs or any other ingredient of the injection solution, a history of malignant, cardiac, psychiatric disorders (including suicidal behaviour), epileptic seizures and infection that may confound the results of the study (such as acquired immunodeficiency syndrome, Hepatitis B or C, upper or lower airway infection) were excluded from this study. MRI-specific exclusion criteria were the presence of pacemaker, inner-ear prosthesis, nerve stimulator, implanted defibrillator, infusion pump, artificial joints or any other metal implant or magnetic or metallic objects that could not be removed from the body (e.g. body piercing, dental prosthesis, implanted electrodes, contraceptive coil, acupuncture needle), claustrophobia, persistent tinnitus, and tattoos or permanent makeup of a particular size (more than 10% of the body surface). Participants were not allowed to take part in other clinical trials with therapeutic intervention during the course of this trial or within one month before enrolment.

**Table 1.** Patient characteristics

Parameter	Total	Avonex	Rebif
Number	*n* = =17	*n* = =8	*n* = =9
Sex (male/female)	7/10	3/5	4/5
Age (yrs)	26.2 (4.9)	25.9 (5.3)	26.4 (4.9)
Paracetamol (yes/no)	11/6	6/2	5/4

The demographic data of the study participants are shown in Table 1. No gender differences in the intake of interferons, paracetamol, age or in the change of STAI or HDRS before and after IFN-beta administration were detected. Similarly, no gender differences in the change of the body temperature were observed. The increase of CXCL10 (*p* = =0.04) following IFN-beta administration was slightly higher for women than for men.

Participants were admitted to the ward on the Clinical Trial Unit in the University Hospital Bonn in the morning of day 1, examined by a physician, and asked for possible concomitant medication. After ECG documentation and the first blood draw subjects received the first subcutaneous Interferon beta application. During 24 h, 5 additional blood samples were taken (timepoints +1, +2, +6, +12, +24 h). The approved standard dose of the drug was administered by trained personnel in prefilled syringes of either Rebif® (Merck, 44 µg IFN beta-1a subcutaneously injected on days 1, 3, 5 and 8) or Avonex® (Biogen, 30 µg IFN beta-1a intramuscular injected on days 1 and 8).

After 24 h in-house stay, participants were discharged for outpatient care returning for the scheduled daily visits. In case of severe side effects (e.g. fever, head and body aches or chills) concomitant medication was administered as foreseen by the study protocol. For monitoring the effect of interferon beta on circulating chemokine levels, plasma levels of C-X-C motif chemokine 10 (CXCL10) were analyzed pretreatment and 6 h after the first administration of interferon treatment

To assess changes in psychopathology, depression and anxiety symptoms were examined with the Hamilton depression rating scale (HDRS, Hamilton, 1960), and the State-Trait Anxiety Inventory (STAI, (Spielberger C., 1967)) prior to the first dose and at day eight after IFN administration. The STAI is based on a self-report consisting of 40 questions with a four-pointed scale and measures both state and trait anxiety. Higher scores are positively associated with more severe levels of depression or anxiety in both questionnaires. Paired *t*-tests by SPSS12 were used to statistically evaluate pre/post-changes in psychometric parameters.

### 2.2 Functional neuroimaging

Functional imaging data were collected on the premises of the German Center for Neurodegenerative Diseases (DZNE, Bonn) using a Skyra 3T MRI scanner (Siemens Healthcare, Erlangen, Germany) by trained personnel. During the scan, participants were continuously monitored by video camera, intercom and manual alarm system. To avoid subject's hearing damages, foam earplugs were applied. Head movements were restrained with foam padding inside a 32-channel head coil. Paradigm pictures were presented on a monitor located behind the scanner visible to the subjects through mirrors. Task responses were recorded by a button-box.

Imaging data were acquired in two scans before and after the eight-days period of administration of recombinant IFN-beta, using a T2*-sensitive echo-planar imaging sequence (TR/TE: 2460/30 msec, flip angle 82°, FOV 24 cm, 64 × 64 pixels of 3 × 3 mm in 39 2.5 mm transversal slices in ascending acquisition order with a gap of .5 mm, giving an isotropic pixel size of 3 mm). To improve sensitivity to signal from basal portions of the brain, including the amygdala, by attenuating susceptibility artefacts, a variable echo sequence was used (Stocker et al., 2006). Echo time was gradually shortened by 8 msec from slice 24 to slice 14, giving a TE of 22 msec in the first 14 slices acquired at the bottom of the volume. Two paradigms were used to assess changes 1. in reactivity to appetitive incentives (targeting the ventral striatum as a key node of the reward system and an anhedonic phenotype) and 2. in amygdala reactivity to negative facial emotional expressions (as a phenotype of an anxious-depressive phenotype). Prior to the presentation of each paradigm, the experimenter communicated briefly with participants to summarize instructions for the task and ensure they had understood them correctly. The two paradigms were presented identically in the scans prior and after treatment. To probe sensitivity to reward cues, a paradigm was employed in which participants could collect money at different amounts (high and low), anticipated by appropriate cues (for a detailed description, see Viviani et al., 2019). Participants were instructed to view the images and to press a button with their thumbs depending on the horizontal position of an appearing dot on the screen. Each correct response was rewarded by appropriate monetary amounts (0.01 € or 0.20 €) and money was paid in cash after the scan (maximal reward: 20.37 €). The reward level was announced by a cue, displayed on the screen for 2 sec, separated by the appearance of the dots by an interval of 3 sec. The dots appeared repeatedly on the screen for about 14.5 sec at irregular intervals following an exponential schedule bound between x and y. There were eight high and eight low reward level trials presented in an irregular sequence. The total duration of this paradigm was 8:07 min. The contrast of interest to assess reactivity to appetitive incentives was the difference in brain signal elicited by cues announcing high and low reward rates (Abler et al., 2006).

To assess amygdala reactivity to emotional stimuli blocks of faces displaying the emotion of anger, sadness, and disgust were displayed in a passive exposure paradigm (Morris et al., 1996; Spohrs et al., 2018a; Stuhrmann et al., 2011). In alternation, blocks of faces and geometric forms were presented resulting in a total scan length of 3:17 min. Reactivity was assessed in the implicit contrast of exposure to faces relative to high-level baseline of geometrical figures, as in previous studies (Dannlowski et al., 2012; Fu et al., 2004; Fu et al., 2008; Harrison et al., 2009; Inagaki et al., 2012; Swartz et al., 2015). Facial emotion images were taken from a set of standardized facial expressions provided by the Swedish Karolinska Institute (Lundqvist et al., 1998).

After realignment and stereotactic normalization into Montreal Neuroimaging Institute (MNI) standardized space (resampling to isotropic voxel size 2 mm with 4th-degree spline interpolation), neuroimaging data were smoothed with a Gaussian kernel (FWHM 8 mm) using the SPM12 package (Wellcome Department of Cognitive Neurology, London: online at http://www.fil.ion.ucl.ac.uk). Regressors for trials at the first level were obtained by convolving an indicator function with a standard blood oxygen level-dependent (BOLD) response curve as provided by the same package. In the reward paradigm, the model included separate regressors for cues and reward events, for the different levels of reward, and for the scan number (at baseline and after the administration of IFN beta). This gave, in each scan, regressors for low reward cue, high reward cue, low reward collection, and high reward collection. In the emotional faces paradigm, the model included a regressor for the time blocks during which participants viewed the facial expressions, giving effects of exposure to faces relative to the implicit baseline of geometric forms. In both cases, the model included the realignment parameters as nuisance regressors and an AR(1) autocorrelation term for the residuals, and was fitted in each voxel separately (Friston et al., 1995). Contrasts of interest at the first level were the interaction between pre/post session and reward level at the cue, and pre/post session and exposure to the emotional facial expressions. The estimated coefficients of the fit from the first level were taken to the second level for group analysis, where a one-sample *t*-test with d.f. = =16 was conducted. At the second level, corrections for multiple testing were obtained using a permutation method as described by Holmes et al., 1996. In brief, the maximum value of the test statistic in the voxels in the region of interest was noted for each of 6000 permutations, and quantiles of this collection of values were used to determine significance levels. For peak-level corrections, the test statistic was Student's *t* in each voxel; for cluster-level corrections, the test statistic was the size of the largest cluster, defined by a cluster-defining threshold *p* < 0.005. The results section reports cluster sizes *k* in number of voxels of 2 × 2 × 2 mm. Correction was obtained for the region of interest in the nucleus accumbens/ventral striatum in experiment by defining a box at MNI coordinates x: −12 ± +12, y: 0 ± +12; z: −12 ± +6, based on the data reported by (Abler et al., 2006). For region of interest analysis of the amygdala, we used maps from the Jülich atlas based on (Amunts et al., 2005). Overlays were produced with the freely available software MRIcron (http://people.cas.sc.edu/rorden/mricron/index.html).

## 3 Results

### 3.1 Psychopathological assessment

**Fig. 1:**
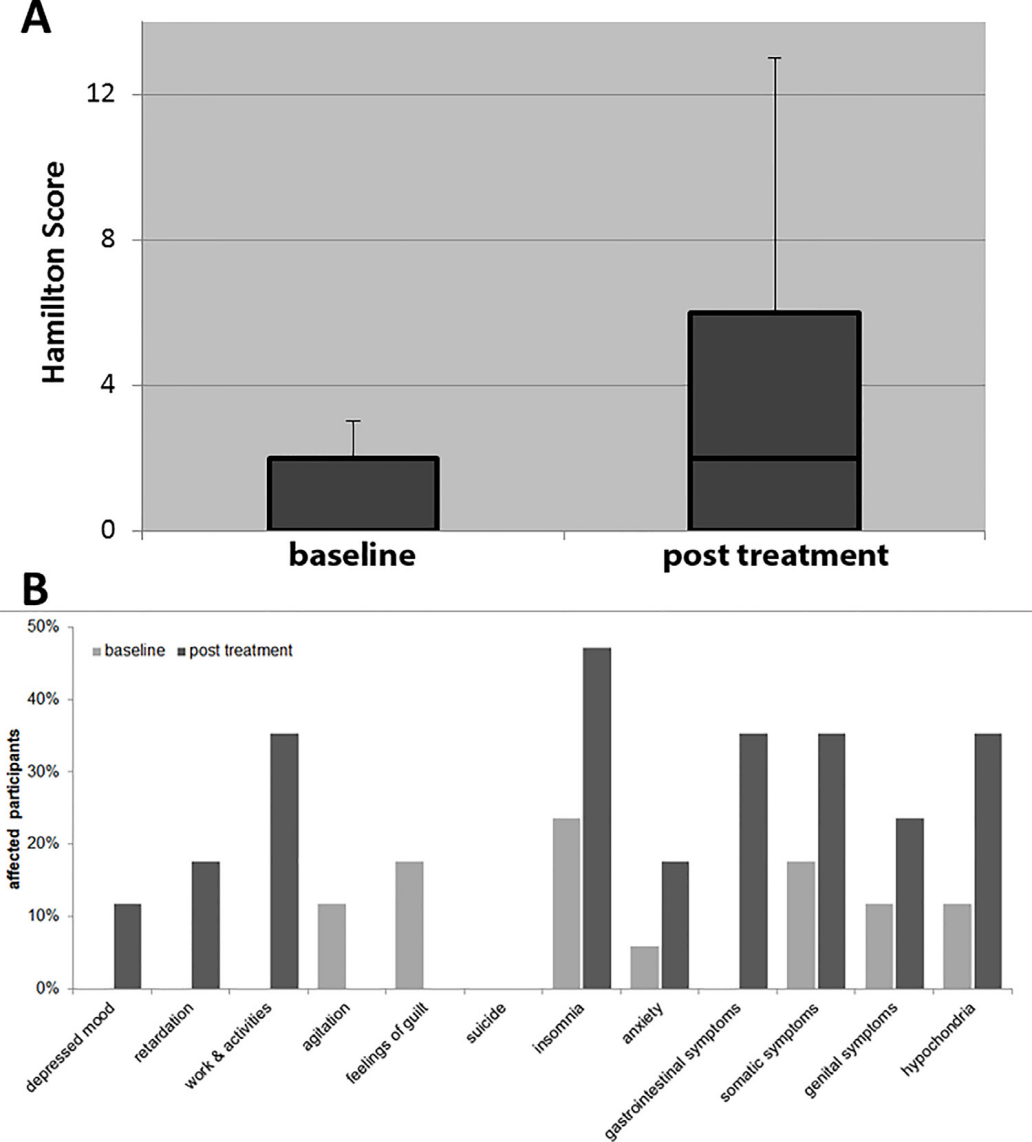
Results of the HDRS before and after IFN-beta. (A) Boxplots showing the median, 75^th^ percentiles and range (antennas). (B) Percentage of participants affected by specific depression symptoms (clustered from single items of the Hamilton depression score) at baseline and after eight days of IFN treatment.

Depression and anxiety were assessed prior and after the eight days of IFN administration with the HDRS and the STAI, respectively (Table 2). The HDRS total score increased over the time with significant changes between baseline and steady-state (*p* = =0.003, Fig. 1A). When analyzing the single items of the HDRS (Fig. 1B), strongest effects were recorded in the items concerning drive and motivation such as: “psychomotor retardation” (*e.g.* slowness of thought or difficulty in concentration), “work & activities” (characterized by feelings of incapacity, fatigue or weakness), “insomnia” (early, middle or late over the night span), “gastrointestinal symptoms” (e.g. loss of appetite or loss of weight) and “somatic symptoms” (e.g*.* indigestion, diarrhea, stomach cramps, heaviness in limbs, loss of energy). Weaker effects were recorded in emotional or physical items “anxiety” (e.g. subjective tension or irritability, worrying about minor matters), “genital symptoms” (e.g. menstrual disturbance, loss of libido) and “hypochondriasis” (characterized by overvalued ides centering on one's health). Two participants complained about depressed mood that occurred after IFN, but no participants reported depressed mood at baseline. In the remaining parameters “agitation” and “feelings of guilt”, no change was observed.

**Table 2.** Treatment effect

Measurements	Group	Baseline/before IFN-beta administration median [range] resp. mean (s.d.)	After 8 days of IFN-beta administration median [range] resp. mean (s.d.)	Statistic (paired *t*-test)	df	*p* Value
STAI	Total Avonex Rebif	30 [25–48] 27.5 [25–40] 32 [26–48]	29 [22–43] 27.5 [22–40] 31 [27–43]	0.69077 0.31399 0.59628	16 7 8	n.s. n.s. n.s.
HDRS	Total Avonex Rebif	0 [0–3] 0 [0–2] 1 [0–3]	2 [0–13] 0.5 [0–4] 6 [0–13]	−3.5193− 1.4331− 3.8733	16 7 8	0.0028 n.s. 0.0047
Body temperature	Total Avonex Rebif	36.7° (±0.3°) 36.7° (±0.3°) 36.7° (±0.3°)	38.7° (±0.4°) 38.6° (±0.5°) 38.8° (±0.4°)	−16.914− 9.7879− 15.222	16 7 8	<0.0001 <0.0001 <0.0001
		Baseline/before IFN-beta administration mean (s.d.)	6 hours after first IFN-administration mean (s.d.)			
CXCL10 (in ng/ml)	Total Avonex Rebif	143.0 (±79.0) 179.9 (±82.9) 110.2 (±62.5)	4543.6 (±1680.6) 3575.1 (±1302.3) 5404.5 (±1546.9)	−10.798− 7.4862− 10.479	16 7 8	<0.0001 0.0001 <0.0001

**Fig. 2:**
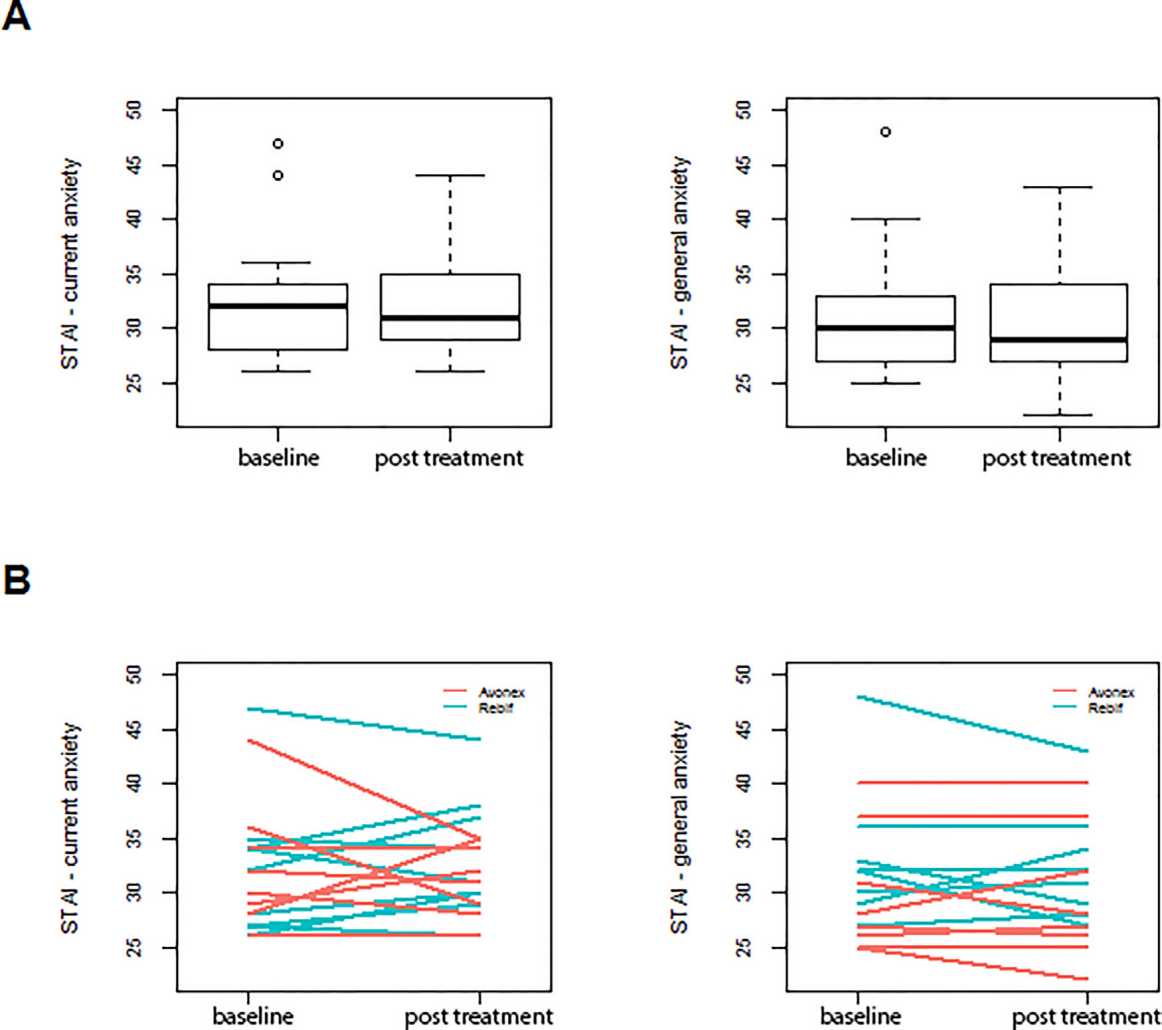
STAI results for 17 participants for current and general anxiety at baseline and after 8 days of treatment with IFN-beta (steady-state condition) are depicted. (A) shows the median of sum scores as boxplot and (B) shows the individual values.

The STAI involved questions about state and trait anxiety. In both parts of this questionnaire no statistical significant differences were observable (state anxiety, *p* = =0.793 and trait anxiety, *p* = =0.351) before (baseline) and after (steady-state) of the eight days treatment period (Fig. 2).

### 3.2 Functional imaging

All participants completed the two tasks, the money-rewarding paradigm and the passive exposure to emotional faces paradigm. In the money-rewarding paradigm, participants could win different amounts of money to probe the impact of IFN-beta on the sensitivity to reward cues (low vs. high) and reward anticipation. Both tasks were administered in the scanner at baseline and after treatment with IFN-beta.

**Fig. 3:**
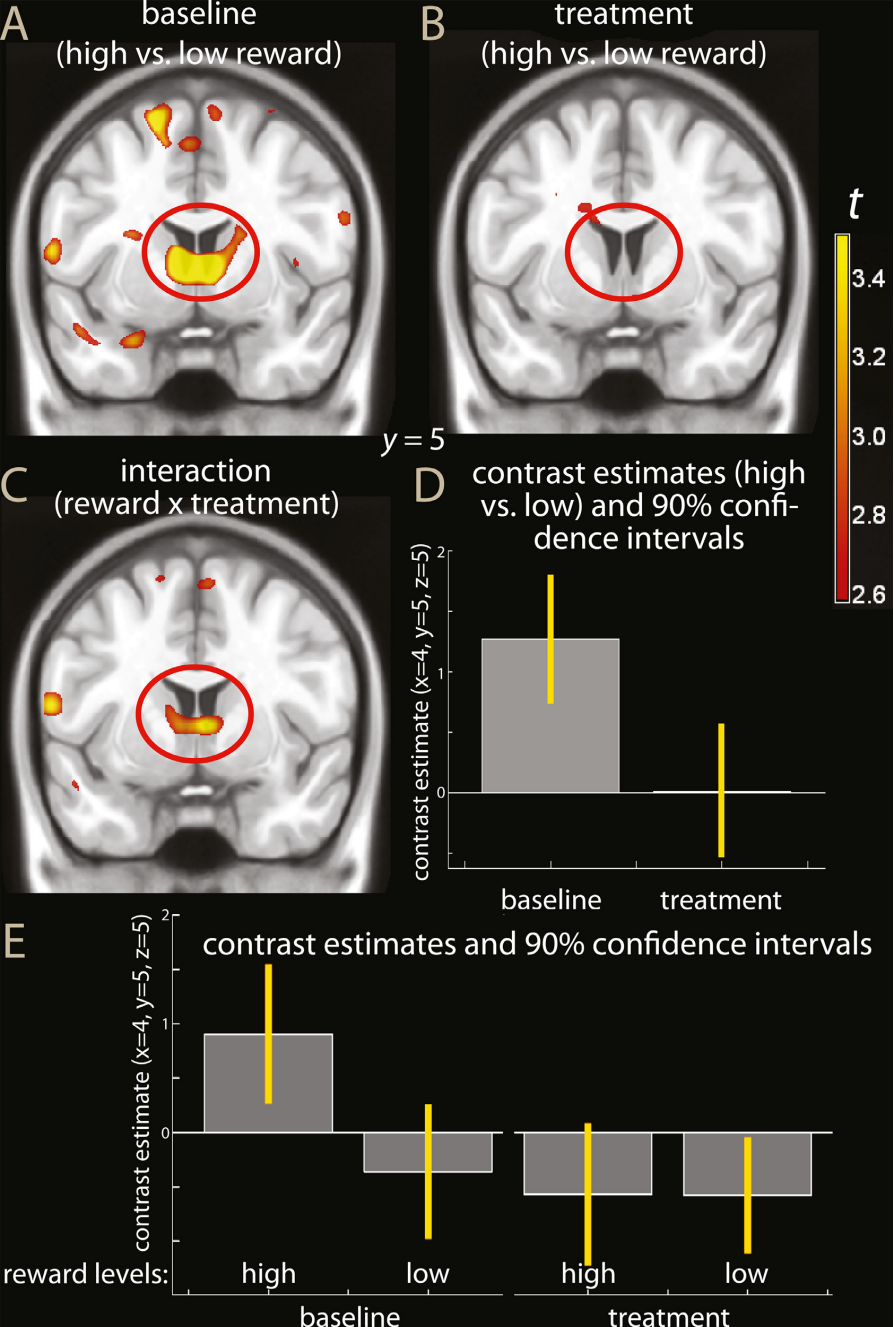
Parametric maps of simple effects of reward (contrast high vs. low reward) in the baseline (A) and treatment conditions (B), and of the interaction high vs. low reward in baseline vs. treatment conditions (C) overlaid on a template brain. The red ovals highlight the effect tested in the region of interest analysis. For display purposes, the maps were thresholded at *p* < 0.005, uncorrected. In D, estimated effects of the contrast high vs. low reward in the baseline and administration conditions (percent signal change). In E, individual effect estimates are shown (high, low: high and low reward trials; baseline, treatment: first and second scan). One can see that the interaction reward levels x treatment was driven by the high reward trials in the baseline scan.

Fig. 3 shows parametric maps of simple effects of reward levels at baseline and after IFN administration and of the pre vs. post administration interaction. Activity of the ventral striatum decreased after IFN-beta in the money-rewarding paradigm (interaction cue high vs. low x treatment: right ventral striatum, MNI coordinates x, y, z: 4, 5, 5, *t* = =3.73, *p* = =0.047, peak-level corrected for the region of interest, and *k* = =145, *p* = =0.012, cluster-level correction; left ventral striatum, −6, 6, 5, *t* = =3.14, *p* = =0.115, peak-level correction, same cluster as above, Fig. 3C and D). The interaction extended posteriorly towards the thalamus (8, −10, 0, *t* = =4.26), however without reaching significance when corrected for the whole brain. As one can see from Fig. 3A and B, the interaction was due to the activation in the baseline condition in the contrast high vs. low reward (−6, 6, 4, *t* = =5.32, *p* = =0.007; 6, 8, 5, *t* = =5.30, *p* = =0.007, peak-level corrected for region of interest) failing to be replicated in the treatment condition (largest activation in region of interest of the same contrast, 6, 4, −8, *t* = =2.52, n.s.).

**Fig. 4:**
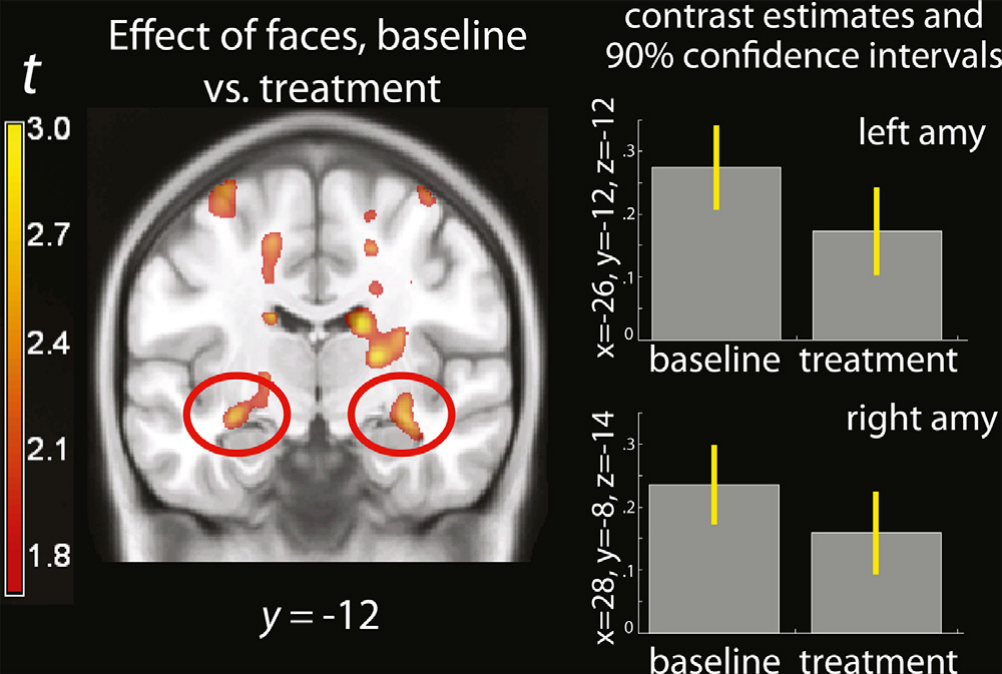
(A) Parametric map of the effect of faces, baseline vs treatment, overlaid on a template brain. The red ovals highlight the effect in the posterior amygdala. For display purposes, the maps were thresholded at *p* < 0.05, uncorrected. (B) estimated effect of exposure to faces with emotional expressions, shown separately in the baseline and IFN-beta administration conditions (percent signal change).

Blocks of emotional faces were displayed in a passive exposure paradigm to evaluate the reactivity of the amygdala to emotional stimuli before and after IFN administration. Here, no significant interaction could be detected between exposure to emotional faces and treatment. As apparent in Fig. 4, the amygdala and the anterior hippocampus were bilaterally activated by the exposure to emotional faces. However, to the extent that an interaction was present in the amygdale, it showed that activity was lower in the treatment than in the baseline condition (−26, −12, −12, *t* = =2.19, *p* = =0.018, uncorrected, Fig. 4).

## 4 Discussion

IFN-beta administration in healthy volunteers not only induced flu-like symptoms but also reduced appetitive motivation in psychopathology ratings. However, there was no impact on negative affect in psychopathological scales. (Capuron et al., 2002; Capuron et al., 2012; Rocca et al., 2007). Consistently with this partial symptom dissociation, IFN-beta treatment was accompanied by a blunted response in the ventral striatum in a task suggesting altered reward system functionality. In contrast, amygdala activity increases when viewing emotionally salient stimuli, commonly associated to affective symptoms of depressiveness and anxiety, were absent.

These findings provide confirmatory evidence that IFN-beta may induce a psychopathological syndrome. Obtained in healthy volunteers, these data are not affected by the possible confound in patient studies introduced by the underlying medical condition, which usually also brings about inflammatory activation or immune changes. While depressive symptoms are known side effects of IFN-beta in patient with multiple sclerosis (MS) (Hoyo-Becerra et al., 2014), it may be difficult to disentangle drug-induced psychopathology from secondary symptoms of the primary pathology. Depression is recorded in about one quarter of all patients with MS, with a lifetime prevalence around 50% (Patten et al., 2003; Patten et al., 2000).

Depression during IFN-beta treatment in MS often leads to treatment cessation and is a cause of non-compliance (Schippling et al., 2016). For this reason, attempts have been made to identify predictors of its occurrence, such as a history of depressive mood and anxiety symptoms prior to treatment (Miller et al., 2014). In a large international cohort study of MS, after exclusion of patients with a previous history of depression, no association between IFN-beta treatment and induction or exacerbation of depressive symptoms was observed (Schippling et al., 2016). However, in the present study, the occurrence of psychopathological symptoms was observed even if participants with a history of depression or depressive symptoms (as assessed by the psychiatric disease diagnostic manual, MINI) were excluded by the admission criteria (Coenen et al., 2015).

Most previous fMRI studies have assessed the effects of IFN-alpha in patient populations (Capuron et al., 2002; Capuron et al., 2012; Rocca et al., 2007), most consistently reporting a blunted fMRI response to reward anticipation consistent with changes in basal ganglia metabolism and dopamine physiology (Capuron et al., 2012). The present study is the first to examine changes in brain activation patterns under the administration of IFN-beta. The effect of the drug was assessed early after the onset of treatment in order to provide information about the mechanism of action of IFN on mood. We evaluated the effect of IFN-beta administration on two MRI phenotypes simultaneously. The amygdala is a limbic structure that is activated by emotionally salient stimuli and shows increased activity in affective disorders such as depression and anxiety, which often accompanies depression. This phenotype is probably best thought of as assessing a transdiagnostic symptom domain of negative affect in predisposed individuals following lifetime exposure to adversity (Whalen et al., 2002). Interestingly, late effects of early adversity have also been associated with increased baseline cytokine levels (for review and meta-analysis, see (Baumeister et al., 2016)). Previous fMRI studies on the effect of inflammation-inducing endotoxins have reported conflicting findings with respect of the reactivity of the amygdala (Harrison et al., 2009; Inagaki et al., 2012). Our findings are rather consistent with an attenuation of amygdala activity, as reported by (Harrison et al., 2009). This finding could also be explained by habituation after repeated exposure to the stimuli. However, no habituation has been observed in work that has examined amygdala response in the contrast to baseline after intervals of days (Johnstone et al., 2005; Spohrs et al., 2018b).

Change in reward circuitries reflecting decreased response to appetitive incentives has frequently been associated with depression (Keedwell et al., 2005; Pizzagalli et al., 2009). Decreased reactivity to reward, withdrawal from social or physical environment, fatigue, and general malaise are common behavioral phenotypes of cytokine-associated sickness behavior (Dantzer et al., 2008; Lasselin et al., 2017). Functional imaging studies in healthy volunteers exposed to endotoxins or vaccination with *Salmonella Typhi* have shown inflammation-dependent reduction of the ventral striatum activity in monetary reward paradigms (Eisenberger et al., 2010). Decreased activation in the ventral striatal region was also shown in a money reward paradigm in patients suffering from hepatitis C being treated with IFN-alpha (Capuron et al., 2012). Furthermore, administration of IFN-alpha in man has also been shown to affect metabolism in the basal ganglia (Capuron et al., 2007) and to have a direct effect on the dopamine system by altering dopamine receptor binding and dopamine turnover (Capuron et al., 2012). These central changes are accompanied by changes in peripheral indices of tyrosine metabolism and CSF dopamine concentrations (Felger et al., 2013).

While the full spectrum of depressive symptoms is listed as a side-effect of IFN therapy (Hoyo-Becerra et al., 2014), fatigue and neurovegetative symptom have been described to develop early. In contrast, anxiety and depressed mood appear several weeks into treatment (Capuron et al., 2002; McNutt et al., 2012). This is consistent with the symptomatic picture recorded in our participants, which were exposed to the drug for only eight days. When analyzing the HDRS ratings, only one subject had a sum score above 8 after IFN, which is usually considered the cutoff to diagnose lower levels of depression. Depressive main symptoms were relatively scarce but neurovegetative symptoms such as feelings of fatigue or weakness, concentration problems, and insomnia were common. These results suggest an induction of sickness behavior by IFN-beta after this short-term treatment period in healthy volunteers but not the full symptomatology of depression.

Interferons are glycosylated hormone proteins with immunomodulatory effects that probably act via receptor-associated tyrosine kinases followed by an activation of the JAK/STAT cascade resulting in an activation of immune cells (e.g. monocytes, leucocytes) as well as an increased expression of human leukocyte antigen molecules and other mediators of immune reactions like cytokines (Pirault et al., 2017). Cytokine-induced sickness behavior is caused by pro-inflammatory events in the brain mediated mainly by the interleukins IL-1a, IL-1b, IL-6, IFN-beta and TNF-alpha (Huang et al., 2008).

Several mechanisms that may explain the behavioral changes induced by IFN have been proposed. One is the activation of primary afferent neural tracts such as the vagal nerve, as commonly observed after infections (Luheshi et al., 2000). A second mechanism is the in an increase in the production of centrally-acting pro-inflammatory peripheral cytokines by activation of macrophage-like cells from the circumventricular organ through activation of a toll-like receptor as part of the humoral pathway response. The circumventricular organ is a specialized part of the brain ventricular system affecting neuroendocrine function (Cottrell and Ferguson, 2004). Yet another possibility is that blood cytokines enter the brain directly either by volume diffusion (Vitkovic et al., 2000) or by active transport mediated by cytokine transporters of the BBB (Banks, 2005). In the brain, they may activate cytokine receptors, which are expressed by both neural and non-neural brain cells (Dantzer et al., 2008; Galic et al., 2012). Cytokines are known to play essential roles in neuroplasticity-associated processes such as neurogenesis or synaptic remodeling (Trollor et al., 2012; Yirmiya and Goshen, 2011) and they are able to counteract and prevent antidepressant actions including effects on neurotransmitter function and synaptic plasticity (Miller et al., 2009). Cytokines may influence neurotransmitter synthesis, release and reuptake and promote depression-like behavior in laboratory animals (O'Connor et al., 2009).

While providing information on the early stages of the psychopathological effects of IFN-beta administration, the present study is affected by several limitations. First, the design did not include a double-blind cross-over procedure. This is only a theoretical possibility in IFN-beta administration, as its side effects are pronounced and obvious to participants as well as to personnel on the ward. Second, treatment was limited to 8 days. It would have been of interest to extend treatment and observe the possible development of a full-blown depressive syndrome. However, ethical constraints precluded this possibility. Finally, our conclusions depend on the imaging phenotypes used here to be equally sensitive and reliable markers of change in the respective symptom domain. In the present study, we adopted these two phenotypes because at present are the most widely used and best understood markers to assess distinct aspects of the psychopathology of affect.

Notwithstanding these limitations, our findings suggest that the mechanisms through which inflammatory processes triggered by IFN-beta induce a psychopathological syndrome may act by first affecting appetitive motivational circuits. This is consistent with symptoms of reduced activity.

There is growing evidence that inflammatory processes may play a role in the pathogenesis of depression, acting in tandem with established psychosocial factors (Baumeister et al., 2016; Hostinar et al., 2018). Symptoms of sickness behavior and depression appear to share common pathogenetic mechanisms (Miller et al., 2013). Therefore, understanding these common mechanisms at both the molecular and phenotypic levels may be of use in identifying patients at risk for affective side effects and developing personalized treatment strategies.
